# Spinal cord compression due to extramedullary hematopoiesis in a patient with E-beta-thalassemia managed without radiation or surgery

**DOI:** 10.1080/20009666.2018.1490141

**Published:** 2018-08-23

**Authors:** Van T. La, Michael Diatte, Johnathan Gaston, Dallas Dick, Raed Sweiss, Zahra Pakbaz

**Affiliations:** a Internal Medicine, University of California, Riverside, School of Medicine, Riverside, CA, USA; b Internal Medicine, Loma Linda University, School of Medicine, Loma Linda, CA, USA; c Neurosurgery, Riverside University Health System Medical Center, Moreno Valley, CA, USA; d Internal Medicine, Riverside University Health System Medical Center, Moreno Valley, CA, USA; e Hematology, Riverside University Health System Medical Center, Moreno Valley, CA, USA

**Keywords:** E-beta-thalassemia, extramedullary hematopoiesis, spinal cord compression, hydroxyurea, blood transfusion

## Abstract

Extramedullary hematopoiesis (EMH) in individuals with thalassemia is often the result of undertreated severe anemia. Radiation or surgery is often the chosen approach to handle spinal cord compression due to these paraspinal EMH elements. Our patient is a 28-year-old male with E-beta-thalassemia who presented with both upper thoracic and lower extremity symptoms of spinal cord compression and was successfully managed with the combination of transfusion and hydroxyurea. Given the variation in symptoms as a result of the sporadic location as well as the extent of these EMH elements along the spinal canal, the hematological communities will continue to benefit from case reports that offer treatment therapy.

## 1. Introduction

Extramedullary hematopoiesis (EMH) is a recognized consequence of undermanaged thalassemia. Spinal cord compression due to these paraspinal extramedullary hematopoietic elements is uncommon and is often managed with either surgery or radiation therapy. We present a case of thalassemia in which these elements were successfully managed by transfusion and hydroxyurea alone.

**Table 1.** Review of literature on treatment methods in cases with similar presentation.

Year	First author	Patient age and gender	Presentation	Location of EMH elements	Management
1992	Konstantopoulos	26 years and Male	Asymmetrical weakness of lower extremities	Thoracic spine	Hypertransfusion to maintain Hgb > 10 g/dL + 2 grams daily hydroxyurea
2000	Bruneteau	19 years and Male	Paresthesia and weakness of lower extremities	Thoracic spine	Transfusion with hydroxyurea

## 2. Case

A 28-year-old Thai male with past medical history of E-beta-thalassemia and splenectomy presented to emergency department with a 3-month progressive tightness in bilateral flank region, weakness, and difficulty walking requiring crutches. Review of system was also significant for unintentional 10-pound weight loss due to poor appetite. Further review of history revealed that he was diagnosed with E-beta-thalassemia at the age of 13 months old. The condition was managed with intermittent transfusion, deferoxamine for iron overload, and hydroxyurea until he turned 21 years old when he stopped following up with his hematologist. He reported that, since then, his baseline hemoglobin was 6 g/dL. Vital signs at admission were within normal limits. Physical exam was significant for frontal bossing with depression of nasal bridge, bilateral costovertebral angle tenderness, slow broad-based gait with ambulation, decreased light touch sensation of the thorax at the level T7-T10, at left medial thigh, and in bilateral lower extremities below the knees. Further neurological exam revealed knee and Achilles hyperreflexia in addition to positive bilateral Babinski, clonus, and Romberg.

**Figure 1.**
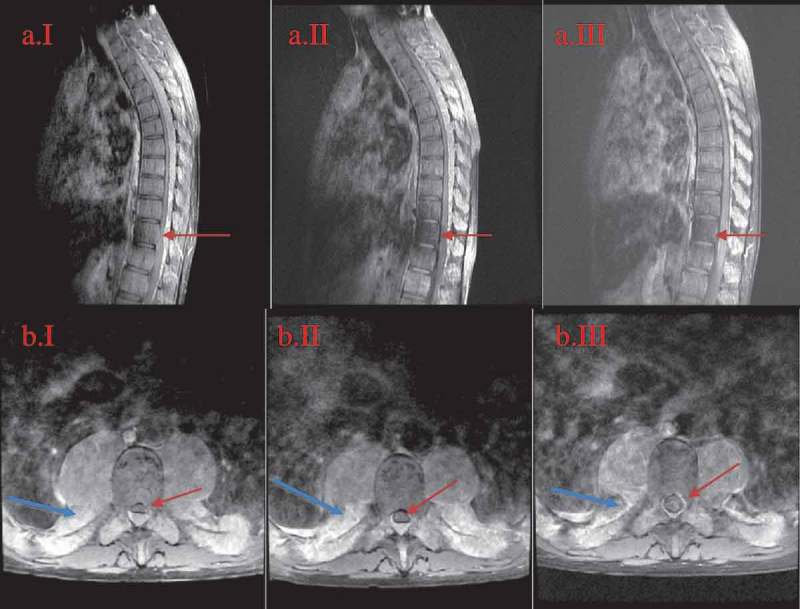
MRI of the thoracic spine in sagittal and axial views at the levels of T6-T7. A = sagittal view; B = axial view; I = initial presentation; II = day 14th after diagnosis; III = week 11th after diagnosis. The regression of the canal stenosis over time from initial presentation to following up can be appreciated on the sagittal view (red arrow) as well as on axial images (red arrow). The reduction of the lesion size can be appreciated on the axial images (blue arrow).

**Figure 2.**
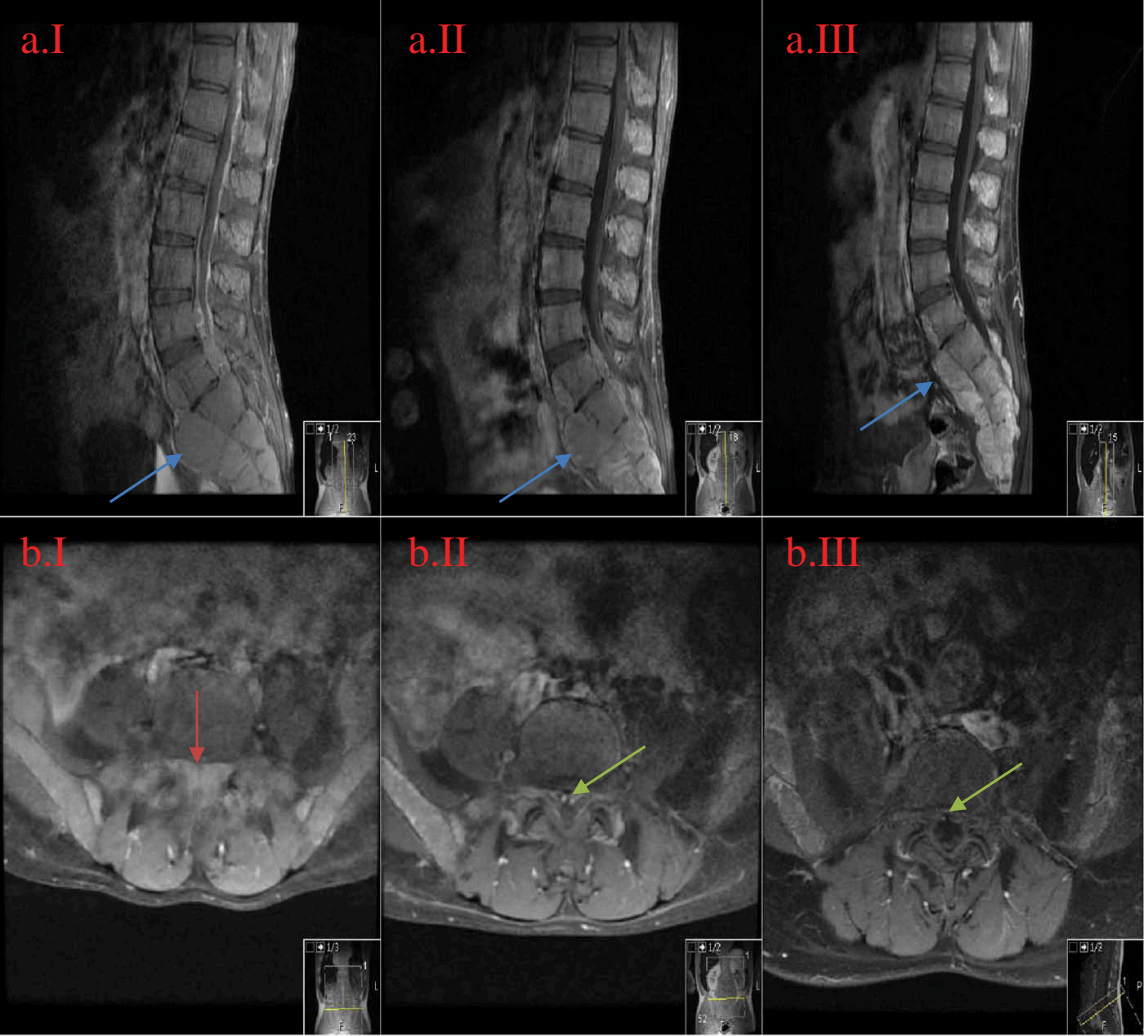
MRI of the lumbar spine in sagittal and axial views at the levels ofL5-S1. A = sagittal view; B = axial view; I = initial presentation; II = day 14th after diagnosis; III = week 11th after diagnosis. The images show regression of the central spinal canal stenosis: from near-complete-obliteration on the initial image (B.II.1 red arrow) to the subsequent re-emergence of the spinal canal (B.II.2 and B.II.3 green arrow) with medical treatment. Also noted is the decrease in size of the bony masses and is the most prominent in the sacral region (II.A images blue arrow).

Laboratory studies were significant for leukocytosis of 72.5 × 10^9^/L, hemoglobin 6.8 g/dL, platelet 732 × 10^9^/L, and reticulocyte count 44.59%. Hemoglobin electrophoresis showed fetal hemoglobin of 49% and hemoglobin E of 59%. Total bilirubin was elevated at 5.3 mg/dL; the rest of the comprehensive metabolic panel was otherwise unremarkable. Further investigations showed that zinc, copper, folate, and vitamin B12 levels were within normal limits. Two-view chest, kidney/ureter/bladder, thoracic, lumbar, and pelvic spine X-rays showed prominence of ribs anteriorly, hepatomegaly, right paraspinal soft tissue prominence surrounding mid-thoracic spines, degenerative joint disease with osteopenia. Magnetic resonance imaging (MRI) of the brain with/without contrast did not show any acute findings but did demonstrate diffuse enhancement involving the clivus and medial occipital bone with mass extension into the bilateral maxillary/sphenoid sinuses, posterior aspect of ethmoid cells, bilateral maxillary bones, inferolateral orbits, and parietal calvarium. MRI of the thoracic spine with/without contrast showed extension of enhancing masses into the spinal canal at the levels of T2-T12 (Figure 1). MRI of the lumbar spine with/without contrast showed prominent medullary expansion of the visualized bony pelvis with associated extension of enhancing mass into the lumbar spinal canal at L5 level causing severe central spinal stenosis and neural foramen narrowing at levels of L5-S1 (Figure 2). These masses were associated with intermediate increased T1 and T2 signal mass-like abnormality suggestive of EMH.

Given the history of untreated chronic severe anemia in the past 8 years, the current physical exam, abnormal laboratory findings, and imaging evidence of paraspinal masses, it was concluded that the masses were extramedullary hematopoietic elements without the need of biopsy risking excessive bleeding. Given the extent and the chronicity of cord compression and severe anemia, the neurosurgical team agreed with hematology to initiate medical management rather than surgical intervention or radiation. Therefore, packed red blood cell (PRBC) transfusion was initiated to gradually increase baseline hemoglobin to above 10 g/dL along with hydroxyurea 15 mg/kg/day. Hydroxyurea dose then gradually was increased to 2000 mg daily to achieve maximum possible hematopoiesis suppression. Surprisingly, 2 days later, the patient regained gross sensation in his lower extremities. After 1 week of hospitalization, gait was noted to be increasingly sturdy, clonus and Romberg improved, and gross sensation of the thoracic area returned. The patient subsequently graduated from physical therapy. By hospitalization day-14 when he was discharged, the patient received a total of 6 units of PRBC. Hydroxyurea was maintained at 2000 mg daily without causing neutropenia or thrombocytopenia. MRI of the thoracic/lumbar spine repeated 2 weeks after the initial imaging showed significant reduction in mass effect on spinal cord and decreased spinal canal stenosis (Figures 1 and 2). After discharge, he was maintained on hydroxyurea 2000 mg daily with blood transfusion to maintain hemoglobin above 10 g/dL. Seven weeks after discharge, neurological symptoms were completely resolved except for patellar hyperreflexia. At this point, hydroxyurea was discontinued due to recurrent pancytopenia. Nine weeks after discharge, repeat MRI of the thoracic/lumbar spines showed no cord compression or spinal stenosis (Figures 1 and 2).

## 3. Discussion

EMH is a physiological compensatory mechanism which occurs when bone marrow cannot meet the circulatory needs. Therefore, EMH occurs in patients with thalassemia whose chronic severe anemia has not been corrected by blood transfusion. Typical sites of EMH can be any organ that participates in hematopoiesis during fetal development such as the spleen, liver, kidneys, and lymph nodes as well as other less commonly known sites such as heart, breasts, prostate, broad ligaments, pleura, cranial nerves, and the spinal canal [1,2]. Particularly, in patients with thalassemia, paraspinal involvement is seen in approximately 15% of the cases [3]. Clinical presentation of EMH elements can range from an incidental finding during the workup for the anemia, to paresthesia/paraplegia/other neurological presentations as in our patient due to spinal cord compression, hemoptysis if pulmonary tissues are involved, cardiac tamponade due to massive pericardial effusion, renal dysfunction, or mistakenly as neoplasm of any organ [1]. The diagnosis of EMH involving the paraspinal tissue/spinal cord compression is best made based upon the combination of history of thalassemia with undertreated severe anemia and isodense to hyperdense T1-T2 weighted findings on MRI with gadolinium contrast preparation [2,3].

In review of literature, radiation therapy and/or surgery are the more commonly used treatment for spinal cord compression from EMH than either monotherapy or a combination of hypertransfusion and hydroxyurea [2–4]. Based on the review of previous reported symptomatic cases of spinal cord compression, depending on the severity of neurological deficit and spinal involvement, most were treated surgically followed by optional radiation [1]. Both surgery and radiation were usually well tolerated by patients whose spinal involvement was not as extensive as this case. Resection of the mass can lead to quick decompression [1]. EMH tissues are highly responsive to low-dose radiation 750–3500 cGy with reduction in volume as much as 16.4% after a treatment course [2,3]. Nonetheless, it was also reported that recurrence rate with radiation therapy or surgery can be as high as 19.1% [2], possibly due to the underlying pathophysiology of uncorrected anemia. Cytopenia is also a concerning complication of radiation while severe bleeding can occur with surgical intervention if the volume of EMH elements is extensive as in this case. Therefore, we chose medical management and postponed surgical approach or radiation.

Monotherapy with transfusion resulted in controversial outcomes. A case reported by Cario et al. showed no significant improvement in EMH size after transfusion [5]. In another case [6], neurological symptoms actually worsened after blood transfusion. It was previously used in cases where radiation was contraindicated such as in pregnancy [7]; and as adjunct therapy when improvement in neurological symptoms was transient and slow [2]. The successful use of hydroxyurea as monotherapy has been reported in the management of EMH masses [8–10]. These patients either had autoimmune hemolytic anemia limiting the use of transfusion [9], hemoglobin level was above 7 g/dL [11], hemoglobin level was at baseline [8], or clinical presentations involving sites other than the spinal cord lessening the urgency of mass shrinkage [12]. Hydroxyurea monotherapy was also the treatment of choice in a patient with cirrhosis when there was a concern for volume overload from transfusion [13], but the response was short lived. We decided on the combined therapy of transfusion and hydroxyurea for our patient as his symptomatic severe anemia warranted the urgency of initiating blood transfusion. Furthermore, the use of hydroxyurea as a monotherapy in this case could have resulted in the drop of hemoglobin or a maximum rise in hemoglobin by 2 g.

Through extensive literature review, we identified two cases in which the combined therapy of transfusion and hydroxyurea were used (Table 1). Compared to these reports, not only the extent of the EMH in our case far exceeded what was described previously, our medical management also resulted in speedy recovery of neurological symptoms and resolution of EMH masses. Konstantopoulos et al. reported a 26-year-old male who presented with history of paraparesis and medical noncompliance as our patient [14]. Due to the lack of the radiation therapy at the facility and that the patient declined transferring to another medical center, a simultaneous therapy of hypertransfusion and hydroxyurea 2 g per day was started. The thoracic extramedullary masses were decreased in size with neurological improvement after 3 months of treatment. Similarly, Bruneteau et al. reported a case of EMH presenting with spinal cord compression whose extramedulary elements were also located only in the thoracic spine [15]. With a combined therapy of transfusion and hydroxyurea, neurological symptoms improved and EMH size was significantly reduced after 2 months of treatment. Given the unusual involvement of both thoracic and lumbar spinal compression in our patient (which is probably due to severe anemia for a long period), we are privileged to report the speedy recovery of the neurological symptoms with a combined therapy of hydroxyurea and hypertransfusion.

Due to its relative rarity and variation in the extent of the EMH elements and clinical presentation, standard of care to manage spinal cord compression due to extramedullary hematopoiesis in patients with undermanaged thalassemia has yet to be established. Until any guideline is available to direct management, the hematological communities will continue to benefit from case reports that offer treatment therapy of EMH elements of unusual location as well as the outcome of such therapy.
